# Analyzing Sleep and Gait Dynamics through Digital Monitoring across Mild Cognitive Impairment (MCI) and non‐MCI Groups

**DOI:** 10.1002/alz.086673

**Published:** 2025-01-09

**Authors:** Minzae Kim, Spencer Low, Preeti Sunderaraman, Rhoda Au, Ludy Shih

**Affiliations:** ^1^ Boston University Chobanian & Avedisian School of Medicine, Boston, MA USA; ^2^ Davos Alzheimer's Collaborative, Philadelphia, PA USA; ^3^ Department of Public Health, School of Public Health, Boston University, Boston, MA USA; ^4^ Framingham Heart Study, Boston University Chobanian & Avedisian School of Medicine, Boston, MA USA; ^5^ Slone Epidemiology Center, Boston University Chobanian & Avedisian School of Medicine, Boston, MA USA; ^6^ Department of Anatomy and Neurobiology, Neurology and Medicine, Framingham Heart Study, BU Alzheimer's Disease Research Center, Boston University Chobanian & Avedisian School of Medicine, Boston, MA USA

## Abstract

**Background:**

Sleep apnea and insomnia are risk factors for dementia. Slower gait and increased gait variability are also associated with increased risk of MCI, linked to cognitive decline. Wearable digital sensors can serve as vital tools for measuring sleep and motor function. While poor quality of sleep has been correlated with decline in cognitive function, the relationship between sleep quality and gait parameters is less characterized. We hypothesized sleep apnea and lower sleep quality were associated with gait speed, gait variability, and functional mobility.

**Method:**

Participants aged 50+ (n=55, female=31) used a finger‐worn sensor with embedded plethysmography [Sleepimage] for at least three consecutive nights over two weeks, quarterly for eight quarters. Five major sleep parameters were evaluated: Sleep Quality Index (SQI), sleep efficiency, Apnea Hypopnea Index (sAHI) at 3% oxygen desaturation threshold, sleep latency, and stable non‐REM sleep percentage. Gait assessments were completed on‐site with inertial sensors [APDM Opals] worn on the wrists, shanks, and waist. It included timed walks with and without cognitive tasks and a Timed Up and Go (TUG) test. Relevant parameters included comfortable gait velocity, dual‐task walking velocity, and lateral step variability. Pearson correlations were used to assess the relationship between sleep and gait parameters. Mean values were compared between participants with and without MCI (MCI=8, Non‐MCI=47) and between slow and fast walkers (above or below 1 meters/second, fast, n=29, slow, n=26) using independent t‐tests.

**Result:**

Mean sAHI was significantly greater in slow (22.39) than fast walkers (14.57, t=2.309, p=0.026). sAHI demonstrated a significant negative correlation with gait velocity (r=‐0.288, p=0.047) and a positive correlation with time required to stand from sitting (r=0.367, p=0.01). Better SQI (r=‐0.316, p=0.029), longer sleep latency (r=0.296, p=0.041), and stable non‐REM sleep (r=‐0.316, p=0.029) was significantly associated with increased lateral step variability. Mean lateral step variability was higher in participants with MCI (16.89) than those without (0.68, t=2.485, p=0.036); mean TUG time was longer in participants with MCI (12.21 seconds) than those without (9.85, t=‐2.755, p=0.028).

**Conclusion:**

This preliminary analysis suggests associations between sleep disturbances and gait parameters associated with risk of cognitive decline. Constraints of sample size and participant diversity are acknowledged, informing the ongoing enrollment of the cohort.

